# Toxicological Effects of Aqueous Extract From African Walnut (*Tetracarpidium conophorum*) Leaves in Rats

**DOI:** 10.1177/2156587217718979

**Published:** 2017-07-18

**Authors:** Seun F. Akomolafe, Ganiyu Oboh, Sunday I. Oyeleye, Tosin A. Olasehinde

**Affiliations:** 1 Ekiti State University, Ado Ekiti, Nigeria; 2 Federal University of Technology, Akure, Nigeria; 3 Federal Institute of Industrial Research, Lagos, Nigeria

**Keywords:** *Tetracarpidium conophorum*, serum biochemistry, histopathology

## Abstract

*Tetracarpidium conophorum* leaves are used in traditional medicine for the treatment of male infertility, without considering its toxicity and side effects. In this study, we investigated the effects of *T conophorum* leaves on some biochemical parameters such as alanine aminotransferase, aspartate aminotransferase, bilirubin, albumin, creatinine, and uric acid. Histology of the liver and kidney were also assessed. The result revealed that the alanine aminotransferase and aspartate aminotransferase levels of the control group were not significantly different from the experimental groups. There was no significant difference in the albumin and bilirubin levels of the control and experimental groups. Similarly, the uric acid and creatinine levels of the experimental rats were not significantly different from the control. The examination of liver and kidney sections did not show any morphological changes and inflammatory cell infiltrations. These findings suggest that the leaves did not induce any pathological changes at the doses tested.

One of the current goals of researchers is to discover new drugs for the development of new products with high therapeutic efficacy and low toxicity profile. To accomplish this, more attention has been drawn to medicinal plants in recent years. Medicinal plants are sources of various chemical substances with potential therapeutic properties.^1^ Medicinal plants are used for the management of diabetes, erectile dysfunction, and cardiovascular, neurodegenerative, and inflammatory diseases.^2^ Unfortunately, most medicinal plants are used indiscriminately without the knowledge of their adverse effects. A previous report has revealed the adverse effects caused by medicinal plants.^3^ These effects could be attributed to the lack of knowledge of the actual dose required for the treatment of diseases.^4^ To optimize the use of medicinal plants, there is a need for thorough investigation of the toxicological effects of these plants at different doses. An approach to determine the toxicity of herbal preparations is to assess their effects on some biochemical parameters and carry out histopathological studies.^5^

*Tetracarpidium conophorum* (Mull. Arg. Hutch & Dalziel), commonly known as African walnut, is a climbing shrub. The plant is locally cultivated mainly for the nuts, which are cooked and consumed as snack.^6^ It is used by elderly people for the treatment of constipation. The bark of the stem is used as laxative and is also chewed to reduce toothache. Odugbemi and Akinsulire^7^ reported that the leaves, stem bark, and fruit of the plant are used for the treatment of toothache, eczema, pruritus, psoriasis, common cold, and prostate cancer. In Nigeria, African walnut is used in folklore for the improvement of male fertility and treatment of dysentery. Previous experimental investigation on African walnut from our laboratory revealed that African walnut leaves contain phenolic compounds such as gallic acid, catechin, chlorogenic acid, caffeic acid, coumarin, rutin, quercitrin, quercetin, kaempferol, and luteolin.^8^

Although Oladiji et al^9^ investigated the toxicological effects of African walnut seeds, to the best of our knowledge, there is little or no report on the toxicity profile of the leaves. Therefore, the present study sought to investigate the toxicological effects of aqueous extract from African walnut leaves using an animal model.

## Materials and Methods

### Materials

#### Plant Material and Authentication

Fresh samples of African walnut leaves were obtained from a farm land near Akure metropolis, Nigeria. The leaves were collected in May 2014, and authentication of the sample was carried out at the Department of Plant Science, Ekiti State University, by Mr Ajayi, and a voucher specimen (Number UHAE 335) was deposited in the herbarium of the same department.

### Sample Preparation

#### Preparation of Aqueous Extract

The leaves were air dried, homogenized, and kept dry in an airtight container prior to the extraction. The plant material (50 g) was soaked in 1 L of cold distilled water for 24 hours. The mixture was then filtered through Whatman No. 1 filter paper and the filtrate centrifuged at 805 × *g* for 10 minutes. The clear supernatant collected was freeze dried and stored in small, capped plastic container at 4°C until required. The plant yield was 12.5 g dry powder/50 g powdered leaf. This was later reconstituted in water for subsequent analysis.

#### Chemicals and Reagents

Methanol, acetic acid, quercetin, and gallic acid were purchased from Merck (Darmstadt, Germany). Sodium carbonate (Na_2_CO_3_), aluminum chloride, potassium acetate, and Folin-Ciocalteu’s reagent were procured from Sigma Aldrich, Inc (St Louis, MO). Isobutyl alcohol, magnesium carbonate (MgCO_3_), iron (III) chloride (FeCl_3_), acetone, chloroform, sodium nitroprusside, methanol, and ferric chloride were source from BDH Chemical Ltd (Poole, England). Water used was glass distilled. Creatinine (Lot No. 315822), aspartate aminotransferase (AST; Lot No. 295800), alanine aminotransferase (ALT; Lot No. 295830), bilirubin (Lot No. 311158), uric acid (UA; Lot No. 309349), total protein (Lot No. 261368), and albumin (Lot No. 302605) kits were sourced from RANDOX Laboratories Ltd, Crumlin, Co, Antrim, UK. Kenxin refrigerated centrifuge model KX3400C was used, while a Jenway UV-visible spectrophotometer (Model 6305; Jenway, Barlo World Scientific, Dunmow, UK) was used to measure the absorbance.

#### Handling of Experimental Animals

Thirty-five male Wistar albino rats weighing between 180 and 250 g were purchased from the Central Animal House, Department of Biochemistry, University of Ilorin, Nigeria. They were housed in stainless steel cages under controlled conditions on a 12-hour light/dark cycle, 24 ± 2°C temperature, and relative humidity of 70 ± 4%. The rats were allowed asses for food and water ad libitum. This study was carried out following approval from the ethics committee on the use and care of experimental animals of the Department of Biochemistry, Federal University of Technology, Akure, Nigeria. The research also adhered strictly to the Principles of Laboratory Animal Care (NIH Publication No. 85-23).

### Toxicity Studies

#### Acute Toxicity Studies

The limit test dose, up and down procedure of the Organization for Economic and Cultural Development^10^ was employed. The extract (3500 mg/kg body weight) was administered separately to 10 rats in a single oral dose and observed for 24 hours. Another group of 10 rats (control) received distilled water. The rats were observed for toxic symptoms such as weakness, loss of appetite, difficulty in movement, reaction to noise, and mortality.

#### Subacute Toxicity Studies

A total of 25 rats were randomly divided into 5 groups of 5 animals each. Animals in groups 2, 3, 4, and 5 were orally administered with a single dose of 500, 1000, 1500, and 2000 mg/kg body weight, respectively, for 28 days. Animals in group 1 were used as the positive control and received distilled water via the same route. The body weights of all the rats were recorded daily throughout the experimental period. After 28 days of treatment, the rats were fasted overnight, anaesthetized with diethyl ether, and sacrificed. Blood samples were collected via cardiac puncture for biochemical analyses.

#### Food and Water Consumption

The amount of food and water consumed were monitored daily as known amount of food and water was given to animal in each cage. After 24 hours, the remaining food and water was taken from the cage and measured. To find out the amount of food and water consumed, the leftover was deducted from the total amount. Food intake was recorded as grams of food consumed/day while water intake was recorded as milliliters of water consumed/day.

#### Body Weight and Relative Organ Weight

The weight of each rat in each group was monitored daily as an index of the physical status of the animals over the period of study. After 28 days, the rats were sacrificed and the liver and right and left kidneys were collected, mopped with filter paper, and weighed. The relative organ weight was calculated and expressed as g/100 g body weight using the following formula:

Relative organ weight = (absolute organ weight g/body weight on sacrifice day g) × 100%

#### Determination of Biochemical Parameters

Albumin was determined according to the method of Doumas et al.^11^ AST and ALT were estimated according to the method of Reitman and Frankel.^12^ Bilirubin (colorimetric method) was carried out according to the method of Jendrasick and Grof^13^ and Sherlock.^14^ Uric acid (colorimetric) was estimated according to the method of Collin and Diehl^15^ and Morin and Proxy.^16^ Creatinine was estimated according to the method of Henry et al.^17^ Protein concentration was determined by the method of Lowry et al.^18^

#### Histopathological Examination

Histopathological examination was carried out using the method of Aliyu et al.^19^ After blood collection, the liver and kidney were carefully dissected from the abdominal region. They were preserved in buffered formalin for 72 hours and then sliced into a thickness of 2.5 mm. The tissues were dehydrated with alcohol of graded concentrations. They were further embedded in paraffin wax and cast into blocks. Sections of the tissues were then cut on a microtome to 5 μm. These sections were later spread onto a slide and allowed to dry. The slides were subsequently stained with hematoxylin-eosin and examined under a light microscope for morphological changes and infiltration of inflammatory cells. Photomicrographs of the samples were taken and interpreted.

#### Phytochemical Screening

The aqueous extract was analyzed according to the methods of Trease and Evans.^20^

#### Determination of Total Phenolic Content

The total phenolic content of the extract was determined using the method of Singleton et al.^21^ Appropriate dilutions of the extract were oxidized with 2.5 mL of 10% Folin-Ciocalteau’s reagent (v/v) and neutralized by 2.0 mL of 7.5% sodium carbonate. The reaction mixture was incubated for 40 minutes at 45°C, and the absorbance was measured at 765 nm in the spectrophotometer (Jenway 6305, Barloworld Scientific). The total phenolic content was subsequently calculated and expressed as milligram gallic acid equivalent/gram dry weight.

#### Determination of Total Flavonoid Content

The total flavonoid content of the extract was determined using a slightly modified method reported by Meda et al.^22^ Briefly, 0.5 mL of appropriately diluted sample was mixed with 0.5 mL methanol, 50 μL 10% AlCl_3_, 50 μL 1 M potassium acetate, and 1.4 mL water, and allowed to incubate at room temperature for 30 minutes. The absorbance of the reaction mixture was subsequently measured at 415 nm, and the total flavonoid content was subsequently calculated using quercetin as standard and expressed as milligram quercetin equivalent/gram dry weight.

### Data Analysis

Data were expressed as mean ± standard deviation (n = 5). The results were analyzed using Student’s *t* test. Post hoc test was also conducted to determine the level of significant difference between each treatment and the control group using Tukey-Kramer multiple comparisons test. Results were considered significant at *P* < .05.

## Results

### Toxicity Studies

**Figure 1.**
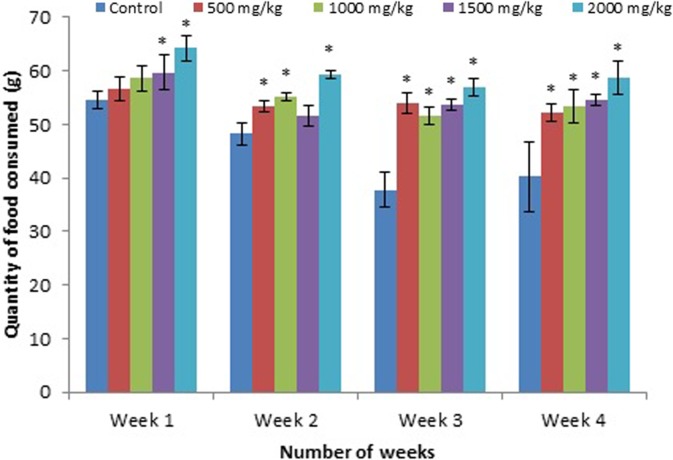
Mean total food intake of rats treated with the *Tetracarpidium conophorum* leaf extract. *Statistically significant from the control.

Table 1 shows the effect of the leaf extract on the body weight of the rats. There were significant (*P* < .05) changes in the mean body weights of the treated groups compared to the control group. Moreover, the treated groups showed a significantly higher weight gain with respect to the dose level. The results in Table 1 show that the increase in the body weights of the rats was relatively dose dependent. Similarly, the total feed intake of the treated groups was significantly (*P* < .05) different from the control, as shown in Figure 1. However, there was no significant change in the relative weights of all the organs of the treated groups compared with the control (Table 2).

**Table 1.** Body Weight Changes of Normal Rats Fed With *Tetracarpidium conophorum* Leaf Extract (g)^a^.

Groups	Week 0	Week 1	Week 2	Week 3	Week 4	Weight Gain
Control	181.00 ± 0.57	182.25 ± 0.50	190.50 ± 0.57	193.50 ± 0.57	200.50 ± 0.57	19
Group 1	181.00 ± 0.57	182.75 ± 0.50	191.00 ± 0.82	198.75 ± 0.96*	211.50 ± 0.57*	31*
Group 2	178.00 ± 0.50	182.00 ± 0.81	201.75 ± 2.63*	210.75 ± 0.96*	213.75 ± 0.96*	35.7*
Group 3	183.00 ± 0.81	191.25 ± 0.50*	202.25 ± 1.71*	212.75 ± 3.20*	220.25 ± 0.50*	37*
Group 4	183.00 ± 0.50	193.00 ± 2.00*	203.25 ± 7.18*	220.25 ± 0.50*	223.75 ± 0.50*	41*

^a^Values represent mean ± standard deviation (n = 5).

*Significantly different from control at *P* < .05.

**Table 2.** Effect of *Tetracarpidium conophorum* Leaf Extract on Relative Organ Weights in Normal Rats (g)^a^.

Organs	Control	Group I	Group II	Group III	Group IV
Liver	2.77 ± 0.33	2.78 ± 0.91	2.76 ± 0.48	2.79 ± 0.11	2.80 ± 0.17
Right kidney	0.40 ± 0.02	0.39 ± 0.12	0.42 ± 0.02	0.41 ± 0.15	0.43 ± 0.01
Left kidney	0.39 ± 0.03	0.41 ± 0.01	0.40 ± 0.02	0.42 ± 0.02	0.43 ± 0.02
Epididymis	0.50 ± 0.03	0.44 ± 0.04	0.46 ± 0.04	0.49 ± 0.03	0.51 ± 0.04
Seminal vesicle	0.27 ± 0.03	0.24 ± 0.02	0.25 ± 0.03	0.26 ± 0.01	0.26 ± 0.03
Prostate gland	0.34 ± 0.01	0.32 ± 0.05	0.32 ± 0.07	0.35 ± 0.07	0.36 ± 0.08
Left testis	0.67 ± 0.01	0.67 ± 0.01	0.68 ± 0.02	0.65 ± 0.01	0.66 ± 0.03
Right testis	0.63 ± 0.01	0.64 ± 0.03	0.65 ± 0.04	0.65 ± 0.04	0.67 ± 0.06

^a^Values represent mean ± standard deviation (n = 5).

*Significantly different from control at *P* < .05.

### Biochemical Parameters

Table 3 depicts the effects of the extract on some liver enzymes. There were no significant (*P* > .05) differences in the concentration of ALT and AST analyzed in the treated groups compared to the control. Furthermore, administration of the leaf extract of African walnut did not cause a significant difference in the concentration of AST and ALT. The extract displayed a hyperproteinemic effect as indicated by the significant increase (*P* < .05) in the total serum protein concentration of all the treated groups when compared to the control group (Table 3). Moreover, albumin levels of the treated groups were not significantly different from the control (Table 3). The serum levels of creatinine and uric acid of the test groups were not significantly different from the control, as shown in Table 3.

**Table 3.** Liver and Kidney Function Parameters of Wistar Rats Fed With *Tetracarpidium conophorum* Leaf Extract^a^.

Parameters	Control	Group I	Group II	Group III	Group IV
Liver function test					
ALT (IU/L)	61.00 ± 3.30^a^	61.49 ± 2.02^a^	60.00 ± 6.91^a^	59.75 ± 7.07^a^	58.80 ± 1.02^a^
AST (IU/L)	204.00 ± 2.07^a^	202.00 ± 3.70^a^	201.00 ± 5.90^a^	200.00 ± 6.16^a^	199.00 ± 8.07^a^
Total protein (mg/dL)	43.86 ± 1.21^a^	49.12 ± 1.20^b^	54.08 ± 2.34^c^	55.37 ± 2.42^c^	57.25 ± 2.53^c^
Albumin (mg/dL)	35.46 ± 0.13^a^	35.23 ± 1.23^a^	35.99 ± 2.10^a^	36.40 ± 1.26^a^	37.00 ± 3.31^a^
Total bilirubin (mg/dL)	11.15 ± 0.51^a^	11.53 ± 0.84^a^	11.56 ± 0.49^a^	12.00 ± 0.04^a^	12.02 ± 0.03^a^
Kidney function test					
Uric acid (mg/dL)	15.95 ± 0.43^a^	15.53 ± 0.97^a^	14.40 ± 1.05^a^	13.63 ± 2.95^a^	13.37 ± 2.97^a^
Creatinine (mg/dL)	0.93 ± 0.01^a^	0.91 ± 0.10^a^	0.90 ± 0.12^a^	0.89 ± 0.10^a^	0.88 ± 0.11^a^

Abbreviations: ALT, alanine aminotransferase; AST, aspartate aminotransferase.

^a^Values represent mean ± standard deviation (n = 5). Values with different superscript letters along the same row are significantly (*P* ≤ .05) different.

### Histopathological Studies

**Figure 2.**
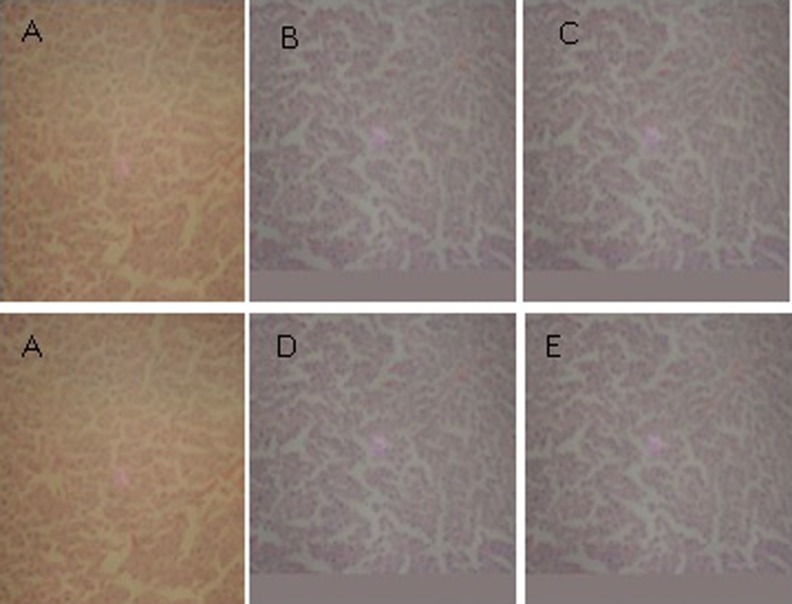
Photomicrograph of the liver of rats treated with the extract of *Tetracarpidium conophorum* (magnification ×200): (A) Control; (B) 500 mg/kg; (C) 1000 mg/kg; (D) 1500 mg/kg; (E) 2000 mg/kg.

**Figure 3.**
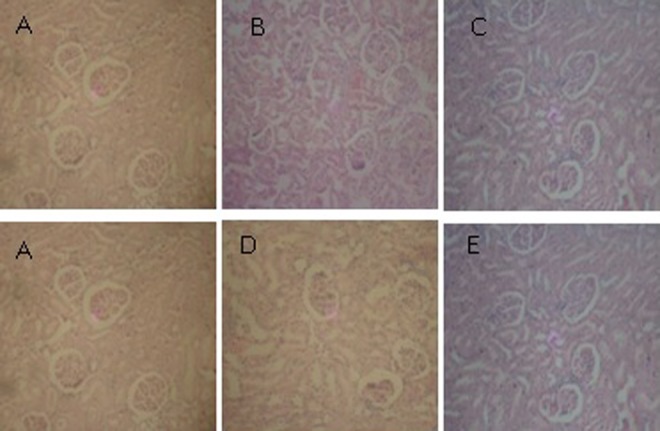
Photomicrograph of the kidney of rats treated with the extract of *Tetracarpidium conophorum* (magnification ×200): (A) Control; (B) 500 mg/kg; (C) 1000 mg/kg; (D) 1500 mg/kg; (E) 2000 mg/kg.

The results in Figures 2 and 3 revealed that there were no pathological alterations in the histological sections of the liver and kidney of rats fed with African walnut leaf extract compared with the control, respectively.

### Phytochemical Constituent

Phytochemical screening of the leaves revealed the presence of saponins, tannin, steroid, flavonoid, terpenoids, and cardiac glycosides as their major secondary metabolites, while alkaloid, phlobatannin, and anthraquninone were absent (Table 4). Furthermore, the total phenolic content (29.31 mg GAE/g) of the extract was significantly higher than its flavonoid content (10.32 mg QE/g).

**Table 4.** Phytochemical Constituents of Aqueous Extract From *Tetracarpidium conophorum* Leaves.

Phytochemicals	Qualitative Screening	Total Phenolic Content (mg GAE/g)	Total Flavonoid Content (mg QE/g)
Saponin	+	29.31 ± 0.47	10.32 ± 0.28
Tannin	+		
Steroid	+		
Phlobotannin	−		
Terpenoid	+		
Flavonoid	+		
Anthraquinone	−		
Cardiac glucosides	+		

+ present; − absent.

## Discussion

Previous experimental investigations have shown the medicinal properties of African walnut in the treatment of different diseases and conditions, especially male infertility. Ikpemel et al^23^ reported that African walnut seed extract improved sperm motility and testosterone and estradiol levels. Previous results from our laboratory also revealed that aqueous extract from African walnut leaf protected rats’ genitals from Fe^2+^-induced oxidative damage.^8^ To the best of our knowledge, there is dearth of information on the toxicological profile of leaves of African walnut.

Our findings revealed that there were significant changes in the body weights of the test groups compared to the control group. This is consistent with previous studies, which revealed that the body weights of animals treated with subchronic doses of aqueous extracts of *Boerhavia diffusa* ^24^ and *Albizzia chevalieri* leaf^25^ increased progressively. Furthermore, we observed that there were no changes in physical activities between the control and the test groups. Moreover, no mortality cases were recorded up to 24 hours post treatment in the test groups. This finding indicates that the extract improved the growth performance of the test groups and had no adverse effect on their metabolic activities. Our observation could also be attributed to the fact that the extract did not induce any pathological changes in the treated rat groups.

The concentration of liver enzymes in the serum is an indicator of hepatic function.^26^ High concentration of AST and ALT indicates tissue damage and altered membrane permeability.^27^ This suggests that the aqueous extract did not induce hepatic damage as no significant difference was observed in ALT and AST levels of the test and control groups. Furthermore, proteins aid the maintenance of osmotic pressure of body fluids and transport of inorganic anions, fatty acids, and drugs.^28^ African walnut leaf extract showed a hyperproteinemic effect as revealed by the increase in total serum protein levels observed in the test groups compared to the control group.

Elevated levels of bilirubin are a common indicator of liver cell dysfunction.^29^ The gradual decrease (Table 3) in the serum levels of bilirubin could be an indication that African walnut leaves did not induce liver damage. The decrease in bilirubin levels allows hepatocytes to conjugate bilirubin. Bilirubin is a useful index of the excretory function of the liver.^30^ In the liver, bilirubin is conjugated with glucoronic acid in a reaction catalyzed by bilirubin-UDP-glucuronyltransferase, which renders it soluble and subsequently excreted into the bile.^31^ Furthermore, serum uric acid and creatinine levels are significant biomarkers that could be used to determine renal function.^32^ In this present study, we observed that there was no significant difference in uric acid and creatinine levels of the test groups compared to the control This result is an indication that administration of African walnut leaves did not induce kidney damage in the rats.

Examination of liver and kidney sections of untreated and treated rats showed no histological abnormalities. Hepatic lobular architecture was normal and infiltration of inflammatory cells was not observed in any of the experimental groups at all the test doses. Hepatic lobular architecture was normal and infiltration of inflammatory cells was not observed in any of the experimental groups at all the test doses. This investigation indicates that oral administration of the African walnut leaf extract did not induce hepatotoxicity and renotoxicity in the experimental rats at the dosage levels tested. This connotes that African walnut leaf could be regarded as safe for medicinal use at the test doses (500 mg to2000 mg/kg) within the period of administration (28 days).

Phytochemical screening of African walnut reveals the presence of saponins, steroids, flavonoids, terpenoids, and cardiac glycosides. This result conforms to what was reported by Obianime and Uche,^33^ but differs from what was reported in the plant seed.^34^ Our findings also revealed that the leaves contain appreciable amounts of phenolic compounds as shown by the total phenol and flavonoid contents. This result correlates with our previous report on the high-performance liquid chromatography phenolic fingerprinting of African walnut leaf.^8^ Previous experimental investigations have shown that these phytochemicals are strong antioxidants and free radical scavengers that can prevent oxidative damage to hepatic and renal cells.^8,34,35^

## Conclusion

This study revealed that aqueous extract from the leaves of African walnut enhances the activities of serum AST and ALT and improves liver and kidney function with no toxicological evidence on the tested organs of the rats. The leaf extract could be used as it contains several secondary metabolites with diverse pharmacological activities. The total phenol and flavonoid content of the leaf extract also revealed the presence of antioxidants. Our findings revealed that the leaf could help in the proper functioning of the liver and kidney. However, chronic toxicity studies on African walnut leaves should be further investigated.
